# Temporal convolutional transformer for EEG based motor imagery decoding

**DOI:** 10.1038/s41598-025-16219-7

**Published:** 2025-09-26

**Authors:** Hamdi Altaheri, Fakhri Karray, Amir-Hossein Karimi

**Affiliations:** 1 Department of Electrical and Computer Engineering, University of Waterloo, Waterloo, ON Canada; 2 Mohamed Bin Zayed University of Artificial Intelligence, Abu Dhabi, United Arab Emirates; 3 Vector Institute for Artificial Intelligence, Toronto, ON Canada

**Keywords:** Transformers, Convolutional neural network, Temporal convolutional network, Grouped query attention, Brain signal decoding, Motor imagery classification, Electroencephalography (EEG), Computer science, Neural decoding, Brain-machine interface

## Abstract

Brain-computer interfaces (BCIs) based on motor imagery (MI) offer a transformative pathway for rehabilitation, communication, and control by translating imagined movements into actionable commands. However, accurately decoding motor imagery from electroencephalography (EEG) signals remains a significant challenge in BCI research. In this paper, we propose TCFormer, a temporal convolutional Transformer designed to improve the performance of EEG-based motor imagery decoding. TCFormer integrates a multi-kernel convolutional neural network (MK-CNN) for spatial-temporal feature extraction with a Transformer encoder enhanced by grouped query attention to capture global contextual dependencies. A temporal convolutional network (TCN) head follows, utilizing dilated causal convolutions to enable the model to learn long-range temporal patterns and generate final class predictions. The architecture is evaluated on three benchmark motor imagery and motor execution EEG datasets: BCIC IV-2a, BCIC IV-2b, and HGD, achieving average accuracies of 84.79, 87.71, and 96.27%, respectively, outperforming current methods. These results demonstrate the effectiveness of the integrated design in addressing the inherent complexity of EEG signals. The code is publicly available at https://github.com/altaheri/TCFormer.

## Introduction

Brain-computer interfaces (BCIs) have emerged as a promising technology that enables direct communication between the human brain and external devices by interpreting neural activity. BCIs hold transformative potential across various domains, from medical rehabilitation to human augmentation, significantly enhancing the quality of life^1,2^. Among various brain signal recording techniques, electroencephalography (EEG) stands out due to its non-invasive nature, low cost, portability, and high temporal resolution, making it particularly suitable for real-world applications. Recent studies have explored EEG-based systems for a wide range of applications, including cognitive skill classification^3^, driver attention monitoring^4^, emotion recognition^5^, and human–robot interaction^6^. In the healthcare domain, EEG plays a critical role in smart healthcare environments^7^ supporting applications such as sleep stage classification^8^ seizure detection^9^ and post-stroke rehabilitation^10^.

Motor imagery (MI)—the mental process of imagining physical movement without actual execution—is one of the most widely used BCI paradigms^1^. It has garnered significant attention due to its potential in neurorehabilitation, assistive technologies, and human-computer interaction. EEG-based MI (MI-EEG) systems have enabled a broad range of applications, including medical uses such as post-stroke rehabilitation^10,11^ prostheses and exoskeleton control^6^ wheelchair navigation^12,13^ and thought-to-text communication^14^. MI-EEG has also been applied in non-medical domains like drone navigation^15^ vehicle control^16^ smart home automation^17^ and virtual reality interfaces^18,19^. However, the real-world deployment of these applications remains constrained by challenges in achieving robust decoding performance and generalization from complex EEG signals^1^.

Decoding motor imagery from EEG signals is challenging due to the low signal-to-noise ratio, which makes it difficult to extract neural patterns associated with specific mental tasks. Additionally, the non-stationary nature of EEG signals, coupled with significant inter-subject and intra-subject variability, adds complexity to the decoding process. Extracting robust and discriminative features from these noisy and variable signals is crucial for accurate motor imagery decoding. Therefore, the development of strong and generalizable models capable of effectively addressing these challenges remains a critical area of research.

Numerous traditional machine learning (ML) and deep learning (DL) techniques have been proposed to address the challenges of decoding MI-EEG signals. Among traditional machine learning approaches that rely on handcrafted features, the Filter-Bank Common Spatial Pattern (FBCSP)^20^ and its variants have consistently demonstrated competitive performance in MI-EEG classification. These methods extract discriminative spatial-spectral features by decomposing EEG signals into multiple frequency bands. Unlike conventional methods, deep learning models can automatically learn rich and discriminative features directly from raw EEG data, eliminating the need for manual preprocessing or handcrafted feature extraction.

In recent years, deep learning-based approaches for MI-EEG classification have made significant progress, leading to the development of various neural architectures. These include convolutional neural networks (CNNs)^21–25^ autoencoders (AEs)^26^ recurrent neural networks (RNNs)^27,28^ temporal convolutional networks (TCNs)^29–32^ deep belief networks (DBNs)^33 ^and more recently, Transformer-based architectures^31,32,34–36^. Among these, CNNs have become the most widely adopted backbone architecture for MI-EEG decoding due to their capability to extract hierarchical spatiotemporal features from raw EEG data^1^.

Both lightweight^24^ and deep CNN models^37^ have been explored for MI-EEG classification, along with various CNN variants such as inception-based CNNs^22,23^ residual CNNs^38^ 3D-CNNs^38^ multi-scale CNNs^25^ multi-branch CNNs^21,38^ and attention-enhanced CNNs^21–23,25,32,39,40^. Notable baseline models include DeepConvNet and ShallowConvNet by Schirrmeister et al.^41^ designed with deep and compact convolutional layers. Another widely adopted model is EEGNet^24^ proposed by Lawhern et al., which utilizes depthwise and separable convolutions to learn temporal and spatial features from EEG data. BaseNet^40^ further combined elements from EEGNet and ShallowConvNet with channel attention mechanisms. While CNNs have shown competitive performance in BCI competitions, their limited receptive field restricts their capacity to model long-term temporal dependencies in MI-EEG data. To address this limitation, recent studies have incorporated temporal modeling components alongside CNNs to enhance their ability to capture sequential patterns and temporal dynamics more effectively^31,32^.

Temporal modeling has been investigated using RNNs and TCNs. For example, the study in^27^ combined a long short-term memory (LSTM) model with FBCSP features and used a support vector machine (SVM) for classification. Another study^28^ employed a gated recurrent unit (GRU) model alongside FBCSP, reporting improved performance over LSTM. As a more recent alternative, TCNs have been proposed for sequence modeling tasks^42^. TCNs utilize dilated convolutions to capture long-range dependencies in time series data while maintaining causality, which is essential for analyzing the temporal dynamics of EEG signals. TCNs offer several advantages over traditional RNNs, including larger receptive fields, reduced parameter complexity compared to standard CNNs, and better training stability due to the absence of vanishing or exploding gradients. These have led to the adoption of TCNs in MI-EEG decoding^29–32^. Ingolfsson et al.^29^ introduced EEG-TCN, a hybrid architecture that combines TCN with EEGNet^24^. Building on this, Musallam et al. further improved the model through multilevel feature fusion^30^. Altaheri et al.^31,32^ proposed an attention-based integration of CNN and TCN modules, achieving superior performance in MI-EEG decoding. Zhao et al.^43^ introduced TCANet, which combines multi-scale CNNs, temporal convolutions, and self-attention to jointly model local and global temporal features, achieving competitive results on BCIC IV-2a and IV-2b with a lightweight design.

Attention mechanisms have been increasingly integrated into deep learning models for EEG analysis to selectively focus on the most relevant information within the data. These mechanisms can adaptively weight the importance of different EEG channels or temporal segments, allowing the model to prioritize task-relevant features and filter out noise. Several recent studies have proposed attention-enhanced deep learning architectures for MI-EEG classification^21–23,25,31,39,40^. For example, Zhang et al.^39^ employed a graph neural network integrated with LSTM and self-attention to classify four MI tasks. Other works introduced hybrid models that integrate inception-based CNNs and LSTMs with attention modules to improve temporal and spatial feature extraction^22,23^. Altuwaijri et al.^21^ developed a multi-branch CNN augmented with squeeze-and-excitation (SE) attention blocks to classify MI tasks directly from raw EEG signals. Wimpff et al.^40^ compared various channel-wise attention strategies integrated with a baseline CNN architecture to evaluate their effectiveness in enhancing the discriminative power of CNN-based models for MI-EEG classification.

More recently, Transformer-based models have opened new possibilities for EEG analysis by leveraging self-attention mechanisms to capture complex dependencies across long sequences. Transformers excel at modeling global relationships within sequential data and can process entire inputs in parallel, offering computational advantages. Their ability to capture long-range temporal dependencies makes them suitable for analyzing the temporal dynamics inherent in EEG signals. However, deploying Transformers directly on EEG data presents several challenges. EEG signals are inherently noisy, and available datasets are typically limited in size, heightening the risk of overfitting for data-intensive Transformer architectures. Moreover, Transformers lack an explicit inductive bias toward locality, which often limits their ability to effectively capture the short-range spatiotemporal patterns that are critical for accurate EEG classification.

To overcome these limitations, recent studies have proposed hybrid architectures that combine CNNs and Transformers by placing Transformer modules after CNN layers^31,32,34–36,44–46^. This design enables CNNs to extract local features while Transformers model global context. Most of these methods adopt a straightforward CNN-Transformer pipeline, where the output of the CNN is directly fed into a Transformer encoder, and the resulting representation is subsequently passed to a classifier. Notable examples of this architecture include EEG Conformer^36^ CTNet^35^ and MSCFormer^34^.

Beyond this sequential integration, other works have introduced customized CNN-Transformer architectures specifically tailored to EEG data. ATCNet^31^ is a notable example that has shown improved performance compared to standard CNN-Transformer pipelines. In ATCNet, a convolutional sliding window is used for patch embedding, and the standard Transformer encoder is modified by replacing the multilayer perceptron (MLP) with a temporal convolutional network, improving the modeling of temporal dynamics. Building on this design, D-ATCNet^32^ incorporates multi-head locality self-attention to enhance spatial awareness and integrated dynamic convolutions to adaptively refine feature representations. However, the use of a sliding window in these architectures increases training time, posing a trade-off between performance and computational efficiency.

Building on recent advancements in hybrid CNN-Transformer architectures, we propose TCFormer, a compact architecture that integrates three core components: a multi-kernel convolutional neural network (MK-CNN) for early temporal-spectral feature extraction, a Transformer encoder with grouped-query attention (GQA) for efficient global modeling, and a temporal convolutional network head for final classification. Unlike prior models that feed Transformer outputs directly to a feedforward classifier, TCFormer fuses the Transformer features with CNN priors and feeds them into the TCN head, which strengthens temporal modeling and improves classification accuracy.

TCFormer enhances both stages of conventional CNN–Transformer pipelines from representational and efficiency perspectives. First, the MK-CNN block improves representational capacity by employing multiple parallel temporal kernels, each specialized in capturing spectral features from distinct EEG frequency bands, mitigating the limited receptive field of standard CNNs. This is followed by a dimensionality reduction step to enhance computational efficiency, and an attention mechanism across kernel groups to emphasize the most informative temporal scales. Second, the GQA Transformer models long-range dependencies more efficiently, reducing memory and computational costs compared to full multi-head attention. To preserve temporal structure, rotary positional embedding (RoPE) is incorporated within the attention mechanism, enabling the encoding of relative temporal relationships without relying on absolute positional indices.

This combination of rich local feature extraction, efficient global context modeling, and long-range temporal decoding enables TCFormer to learn highly discriminative representations from MI-EEG signals. The architecture is designed to be both performance-optimized and computationally efficient, making it well-suited for practical BCI applications.

The main contributions of this paper are summarized as follows:

- Proposes TCFormer, an efficient architecture that integrates multi-kernel convolutions, grouped-query Transformer encoding, and temporal convolutional decoding for EEG-based motor imagery classification.
- Designs a multi-kernel CNN block that captures diverse temporal-frequency EEG patterns through parallel convolutions and grouped attention, enhancing spatiotemporal feature representation.
- Employs a grouped-query attention Transformer with rotary positional embedding to efficiently model long-range dependencies in EEG sequences.
- Integrates a grouped TCN head for causal temporal decoding, maintaining feature group separation and enabling enhanced sequence-level classification.
- The complete codebase, including benchmark comparisons with state-of-the-art methods, is available at https://github.com/altaheri/TCFormer for reproducibility and further research.

The remainder of this paper is organized as follows: Sect. 2 describes the proposed TCFormer architecture in detail. Section 3 presents the experimental results along with a discussion of key findings. Finally, Sect. 4 concludes the paper.

## Proposed method: TCFormer architecture

**Fig. 1:**
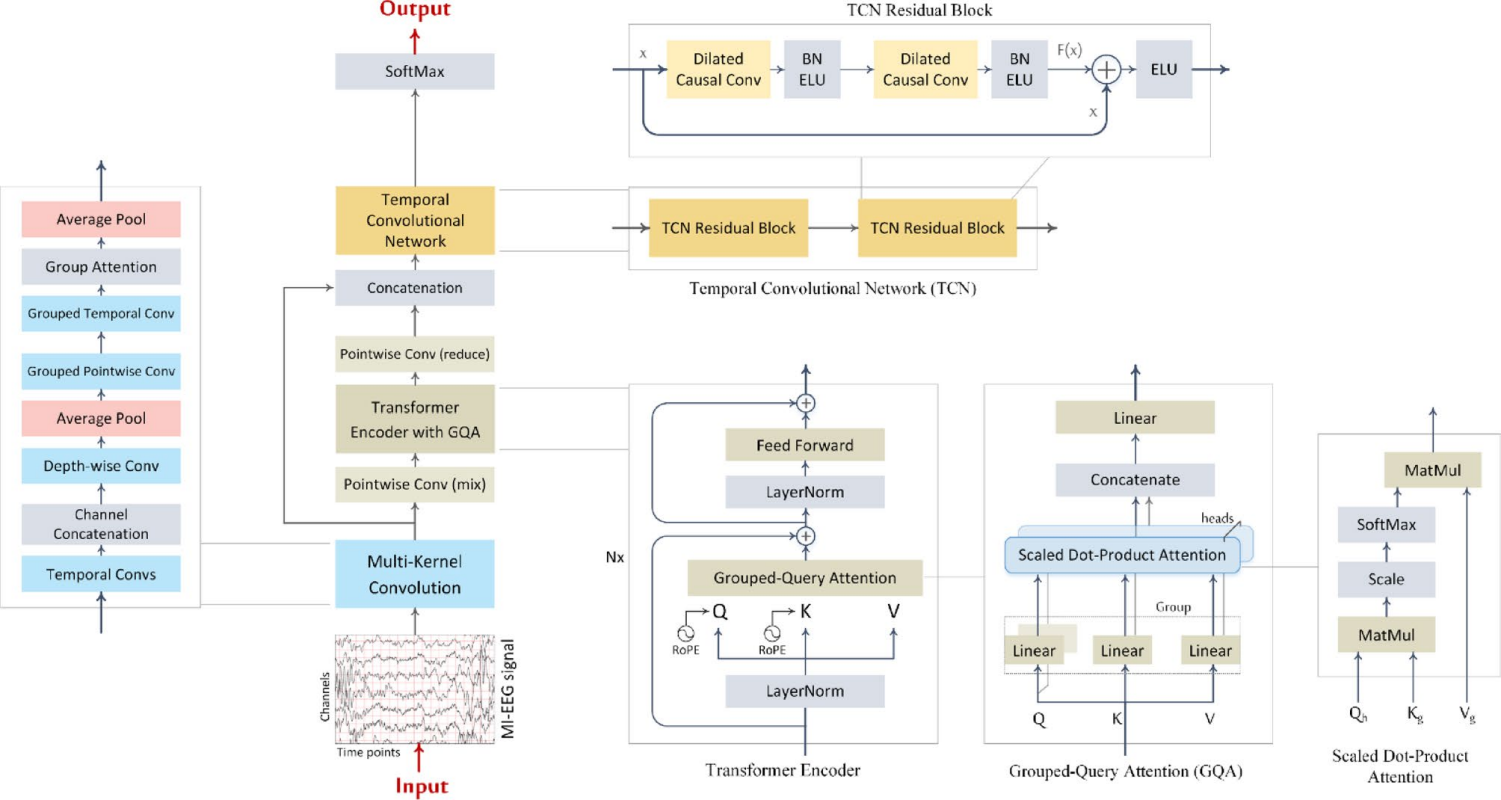
TCFormer architecture, comprising a convolutional module, a Transformer encoder, and a TCN, followed by a classifier. The convolutional module performs multi-kernel temporal filtering and spatial (depth-wise) filtering to extract multi-scale EEG features. The Transformer encoder, composed of $\:N$ stacked layers, applies grouped-query attention (GQA) and feed-forward (FF) sublayers to model global dependencies, with rotary positional embeddings (RoPE) providing temporal context. The TCN module utilizes causal dilated convolutions to capture local temporal patterns and sequential dependencies that are not captured by the Transformer and produce the final class logits. *Conv* convolution, *BN* batch norm, *ELU* exponential linear unit.

The TCFormer architecture comprises three main components (Fig. 1): a multi-kernel convolutional front-end for spatiotemporal feature extraction, a grouped-query Transformer encoder for global sequence modeling, and a temporal convolutional network head for final temporal decoding and classification. The MK-CNN block applies parallel temporal convolutions with varying kernel sizes, depth-wise spatial filtering, and average pooling to extract rich multi-scale spatiotemporal features from raw MI-EEG trials, generating a compact sequence of feature tokens. These tokens are processed by the Transformer encoder, which consists of stacked layers employing grouped-query self-attention and rotary positional embeddings to capture global temporal dependencies efficiently. The context-enhanced representations are then passed to the TCN head, where dilated causal convolutions integrate long-range temporal patterns to produce the final class logits. Each of these components is described in detail below.

### Input representation and preprocessing

Motor imagery EEG decoding is formulated as a supervised multi-class classification problem, defined as follows. Let

$$\:S={\left\{\left({X}_{i},{y}_{i}\right)\right\}}_{i=1}^{m},\:\:\:\:\:\:\:{X}_{i}\in\:{\mathbb{R}}^{C\times\:T},\:\:{\:y}_{i}\in\:\left\{1,\dots\:,n\right\}$$

Where $\:S$ denotes a collection of $\:m$ labeled trials, and each trial $\:{X}_{i}$​ is a matrix of $\:C$ EEG channels recorded over $\:T$ time-samples and $\:{y}_{i}$ is its class label (e.g., *Left hand*, *Right hand*, *Feet*, *Tongue* for $\:n=4$). The learning objective is to estimate a parametric decoder as follows:

$$\:f\left(X;\theta\:\right):{\mathbb{R}}^{C\times\:T}\to\:\left\{1,\dots\:,n\right\},\:\:\:\:\text{s}\text{u}\text{c}\text{h}\:\text{t}\text{h}\text{a}\text{t}\:f\left({X}_{i};\theta\:\right)={y}_{i}\text{}\:\text{f}\text{o}\text{r}\:\text{u}\text{n}\text{s}\text{e}\text{e}\text{n}\:\text{t}\text{r}\text{i}\text{a}\text{l}\text{s}$$

Before training, MI-EEG signals are commonly subjected to preprocessing to enhance signal quality and improve classification accuracy. Traditional approaches often involve artifact removal, channel selection, and bandpass filtering. However, recent studies have shown that deep learning models can achieve competitive or superior performance even when trained directly on raw EEG signals^1^.

In this work, a minimal preprocessing pipeline is adopted to preserve the complete spatiotemporal structure of the EEG data. For all datasets, per-channel standardization is applied to ensure numerical stability during training. Each EEG channel $\:{\text{x}}_{i}$ is normalized as follows:

$$\:{\text{x}}_{i}^{{\prime\:}}=\frac{{\text{x}}_{i}-\mu\:\left({\text{x}}_{i}\right)}{\sigma\:\left({\text{x}}_{i}\right)},\:\:i=\text{1,2},3,\dots\:,C$$

where $\:\mu\:\left({\text{x}}_{i}\right)$ and $\:\sigma\:\left({\text{x}}_{i}\right)$ are the mean and standard deviation of the channel $\:{\text{x}}_{i}$, calculated across all training samples and time points. This standardization is performed using statistics computed from the training data only and then applied consistently to validation and test data.

**Fig. 2:**
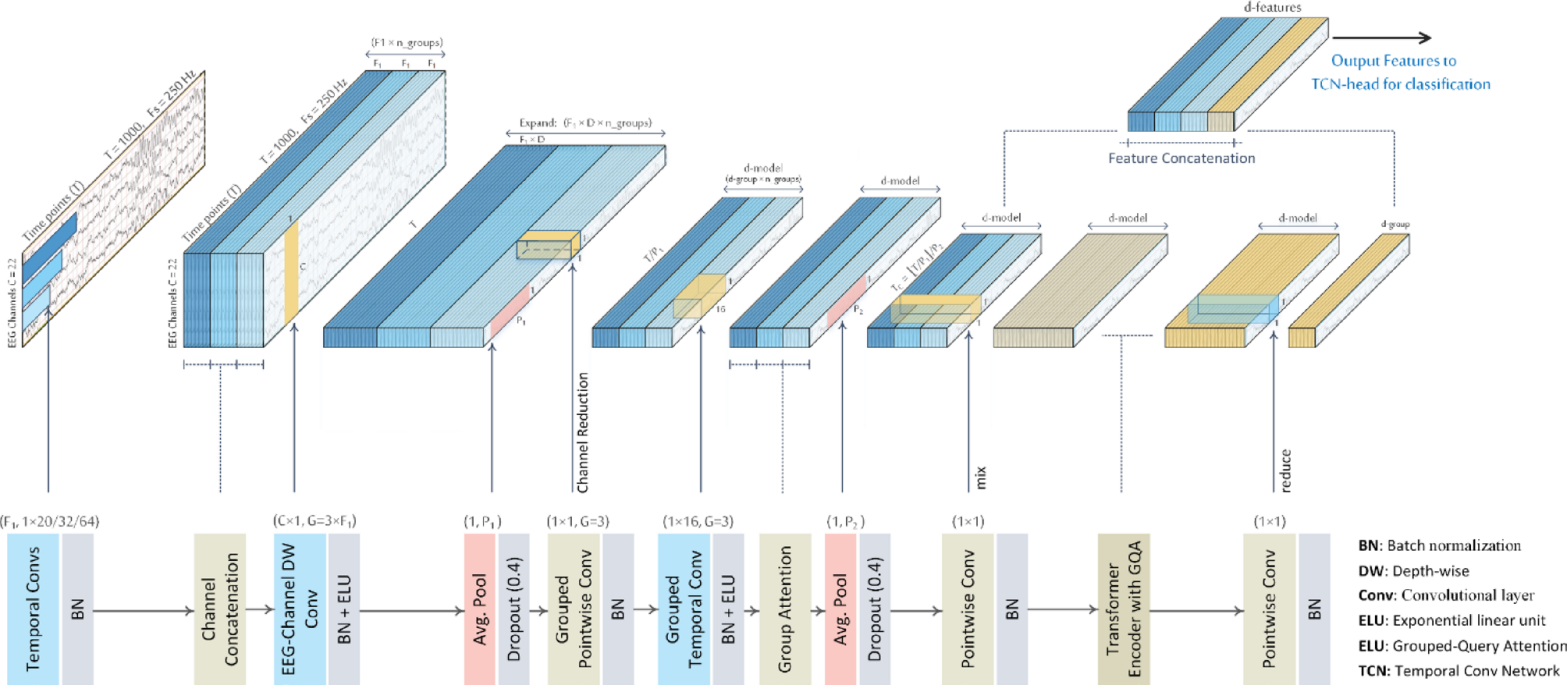
Feature-extraction pipeline: raw EEG is first processed by a multi-kernel convolutional block to capture diverse temporal scales, then passed through a Transformer encoder with grouped-query attention to model global dependencies, yielding the encoded feature tensor that feeds into the TCN classification head.

### Multi-kernel Convolution

The MK-CNN module introduces three key design elements. First, it expands the number of convolutional kernels at the input stage, allowing for the early extraction of rich temporal and spatial features directly from raw EEG signals. This expansion is followed by a channel reduction step, which compresses the feature representation to ensure computational efficiency in the subsequent layers. Second, it utilizes multi-kernel temporal convolutions, where different kernel sizes are used in parallel to capture a broad range of frequency components—shorter kernels are sensitive to high-frequency features, while longer kernels capture low-frequency temporal patterns. Each kernel group is processed independently to preserve distinct temporal characteristics. Third, it applies grouped attention mechanisms across these kernel groups, allowing the model to focus on the most informative features across different temporal scales.

Raw EEG signals are first processed by the MK-CNN block, which generates a sequence of feature “tokens” for the Transformer. Specifically, given an input trial of shape $\:C\times\:T$ (with $\:C$ channels and $\:T$ time points), the MK-CNN module employs multiple temporal convolutions along the time axis using kernel lengths $\:{K}_{c}=\left\{20,\:32,\:64\right\}$, each with $\:{F}_{1}=32$ filters, as illustrated in (Fig. 2). At a sampling rate of 250 Hz, these kernels correspond to temporal windows of 80, 128, and 256 ms, respectively. Each kernel is tuned to a specific frequency band, based on the principle that 1–2 oscillatory cycles should fall within its receptive field for effective temporal pattern recognition. Accordingly, the 20-sample kernel captures β-band activity (13–30 Hz), the 32-sample kernel targets the µ-band (8–12 Hz), and the 64-sample kernel focuses on θ to low-α activity (4–8 Hz), which has also been shown to contain discriminative motor imagery information^1^. Kernels smaller than 20 samples generally fail to capture relevant µ/β components, while those larger than 64 samples tend to respond to low-frequency noise (< 4 Hz). This kernel configuration thus achieves a balance between spectral selectivity and noise suppression.

The resulting feature maps are batch-normalized to stabilize training and then concatenated along the feature channel dimension. Note that in this context, the term channel refers to the feature channel dimension in the CNN representation and should not be confused with the EEG channel dimension, which denotes the number of electrode sensors in the raw EEG signals. Throughout this paper, we consistently use channels to refer to CNN feature dimensions and EEG channels to denote electrode dimensions.

Next, a depth-wise convolution with a filter size of $\:C\times\:1$ is applied across the EEG channels to extract channel-specific features. This operation expands the representation by applying $\:D=2$ filters per feature map, allowing the model to learn discriminative combinations of EEG channels for each time-frequency component. The output consists of $\:{F}_{1}\times\:D$ feature maps, each of temporal length $\:T$.

To reduce the temporal resolution, an average pooling layer with a downsampling factor of $\:{P}_{1}=8$ is employed. This preserves essential information while lowering the temporal resolution from the original sampling rate (e.g., 250 Hz in BCIC-IV) to approximately 32 Hz. A pointwise $\:1\times\:1$ convolution is then applied for channel reduction, projecting the representation down to a fixed dimensionality of $\:{d}_{model}={d}_{group}\times\:\:{n}_{groups}$, where *n*_*groups*_ is the number of temporal convolution branches (e.g., three when *K*_*c*_={20, 32, 64}).

Following this, a second temporal convolution is applied using $\:{F}_{2}={d}_{model}$ filters with a kernel size of $\:{K}_{C2}=16$. This convolution extracts higher-level temporal features over a fixed window (e.g., a 500 ms window at a 32 Hz sampling rate). An average pooling operation with a stride of $\:{P}_{2}=7$ is then applied to further reduce the temporal resolution, resulting in a fixed-length sequence of temporal tokens, denoted as $\:{T}_{c}$​. The total number of tokens is computed as:

$$T_{c} = \left\lfloor {{{\left\lfloor {\frac{{\text{T}}}{{P_{1} }}} \right\rfloor } \mathord{\left/ {\vphantom {{\left\lfloor {\frac{{\text{T}}}{{P_{1} }}} \right\rfloor } {P_{2} }}} \right. \kern-\nulldelimiterspace} {P_{2} }}} \right\rfloor$$

Where $\:\text{T}$ is the original input length (i.e., the number of EEG time points). Each resulting token is a feature vector of dimension $\:{d}_{model}=48$, encoding multi-scale temporal and spectral information across EEG channels.

#### Grouped squeeze-and-excitation (SE) attention

**Fig. 3:**
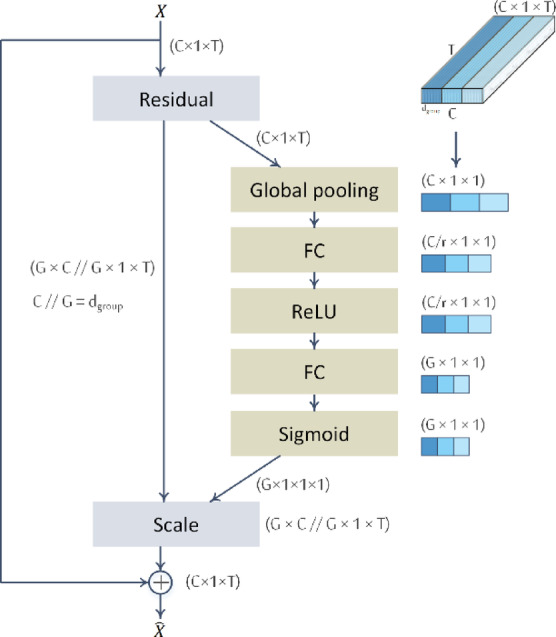
Grouped squeeze-and-excitation (SE) attention in the multi-kernel convolution block. The input feature map $\:X\in\:{\mathbb{R}}^{C\times\:1\times\:T}$ is globally average-pooled along the temporal dimension to produce a channel descriptor of shape $\:C\times\:1\times\:1$. This descriptor is passed through two $\:1\times\:1$ grouped convolutions, with reduction ratio $\:r$, followed by ReLU and sigmoid activations, resulting in $\:G$ per-group attention weights. These weights, each in the range $\:\left[0,\:1\right],$ are broadcast across their corresponding $\:C/G$ channels and used to rescale the original input. The reweighted features are then added to the residual path to produce the final output $\:\widehat{X}$.

To enhance temporal feature representations, we integrate grouped Squeeze-and-Excitation (SE) attention within the multi-kernel convolution block, as illustrated in (Figs. 1 and 2). Grouping is performed along the channel dimension, with each group corresponding to one of the temporal kernels in $\:{K}_{c}=\left\{20,\:32,\:64\right\}$, resulting in three channel groups. Attention weights $\:{a}_{j}$ are computed using the SE mechanism^47^ which follows the standard three-step process (squeeze, excitation, and scaling), but with the excitation stage producing $\:G$ group-wise weights instead of $\:C$ channel-wise weights, as shown in (Fig. 3).

Specifically, global average pooling is first applied over the temporal dimension to produce a single descriptor per channel. These descriptors are then passed through a grouped 1 × 1 convolution to project them into a lower-dimensional space ($\:C/reduction$) within each group, followed by a ReLU activation. A second grouped 1 × 1 convolution reduces each group’s representation to a single scalar attention weight $\:{a}_{j}\in\:[0,\:1]$, normalized via a sigmoid function. The resulting attention weights are broadcast across all channels in their respective groups and used to reweight the feature maps. This mechanism enables the model to dynamically emphasize or suppress the contribution of each temporal scale, improving its ability to focus on task-relevant temporal dynamics.

### Transformer encoder with grouped-query attention

The Transformer encoder comprises $\:N$ identical layers, each consisting of a grouped-query self-attention module^48^ with rotary positional embeddings^49^ followed by a position-wise feed-forward network (FFN). Residual connections are applied around both sub-layers, with layer normalization following a pre-norm configuration, as illustrated in (Fig. 1).

**Fig. 4:**
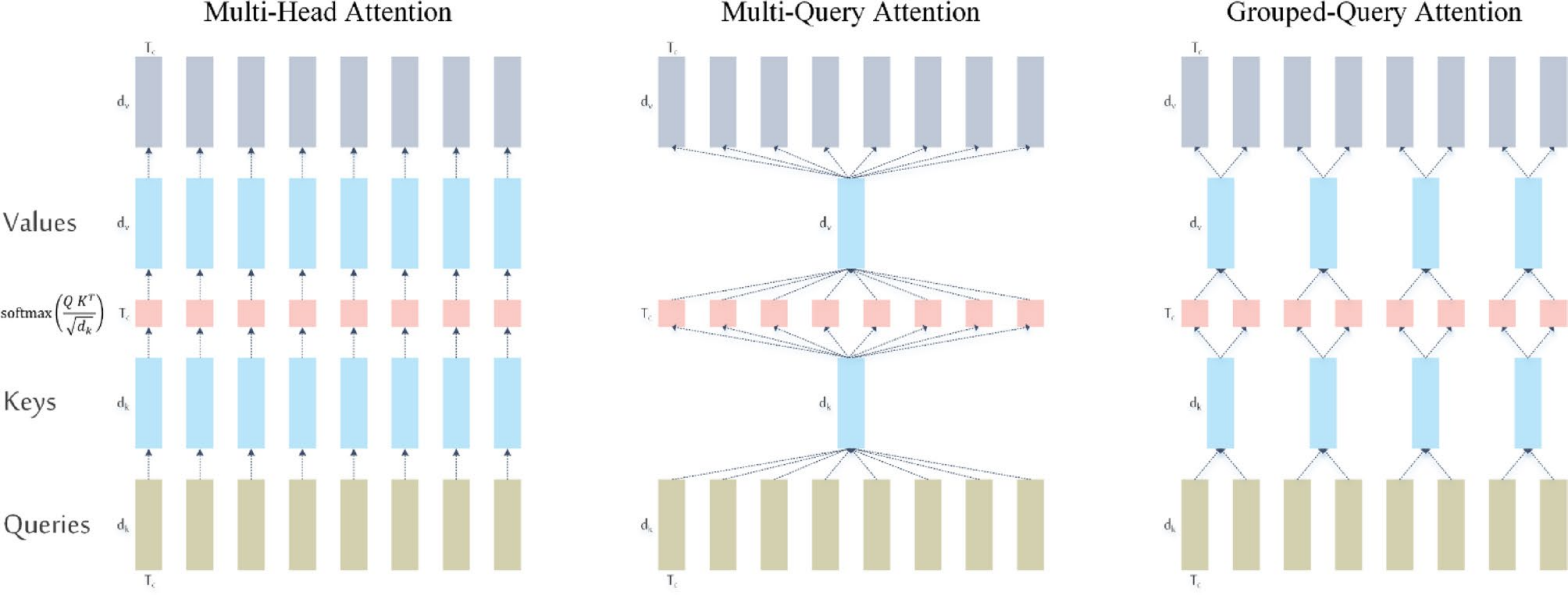
Comparison of attention mechanisms. (Left) multi-head attention (MHA): each query head has its own key- and value-projection matrices. (Middle) multi-query attention (MQA): all heads share a single key-value pair, minimizing memory cost. (Right) grouped-query attention (GQA): query heads are divided into groups, with each group sharing a key-value pair—providing a trade-off between the expressiveness of MHA and the efficiency of MQA. GQA reduces to MQA when $\:G=1$, and becomes equivalent to MHA when $\:G=H$.

**Fig. 5:**
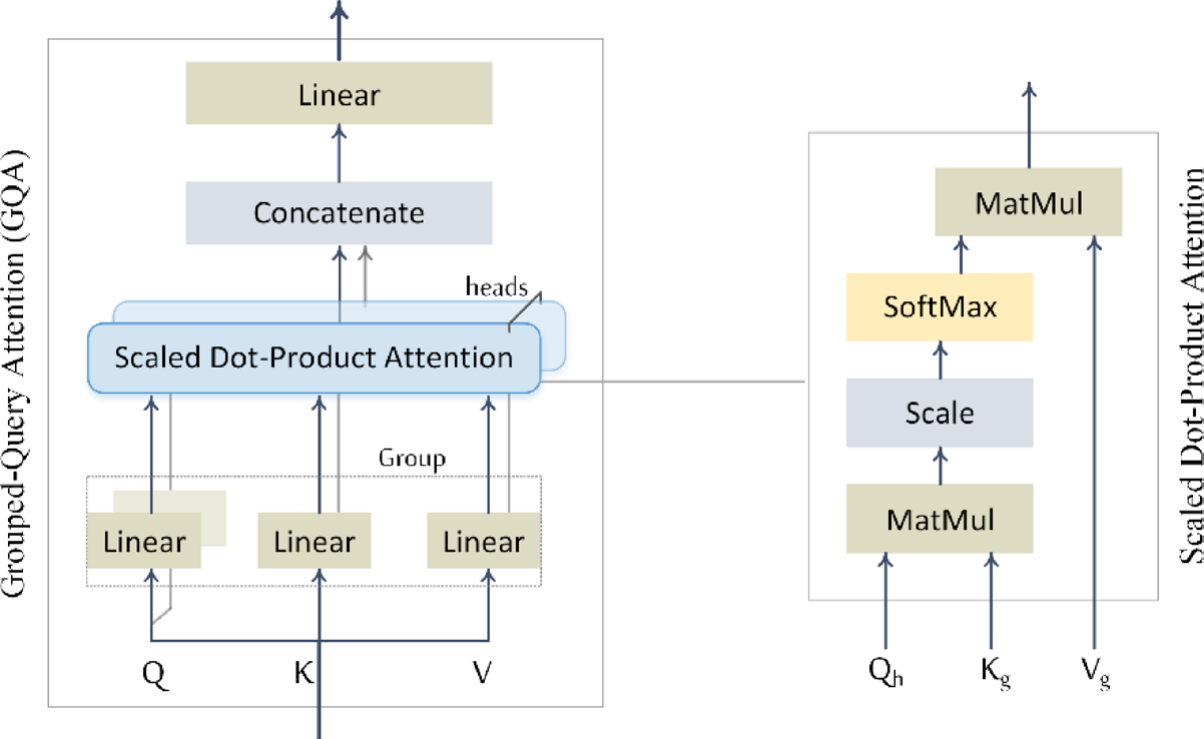
Grouped-query attention (GQA).

Grouped-query attention is adopted as an efficient self-attention mechanism that balances the expressive capacity of Multi-Head Attention (MHA)^50^ with the computational efficiency of Multi-Query Attention (MQA)^51^. GQA generalizes both MHA and MQA by introducing an intermediate number of key/value groups. Specifically, GQA divides the $\:H$ query heads into $\:G$ groups, where $\:1<G<H$. Within each group, all query heads share a single key and value projection matrix, while maintaining distinct query projections, as illustrated in (Fig. 4). When$\:\:G=1$, GQA reduces to MQA, where all heads share the same key-value pair. When $\:G=H$, it becomes equivalent to standard MHA, where each head has its own distinct key and value projections. In our implementation, we set $\:G=H/2$ to achieve a compromise between representation capacity and computational cost. As shown in Fig. 5, GQA retains the core MHA components—linear Q/K/V projections, scaled dot-product attention, and output projection—while reducing redundancy by sharing key/value projections across groups.

Positional information is embedded using Rotary Positional Embedding, replacing traditional fixed sinusoidal or learnable positional encodings. RoPE introduces position-dependent rotations to the query and key vectors, enabling the model to capture relative positional relationships directly within the attention mechanism. This approach has been shown to improve the modeling of temporal dependencies^52^. In the proposed architecture, RoPE is applied within each attention layer as part of the GQA computation.

The following presents the mathematical formulation of the proposed Transformer encoder with GQA. Given an input sequence $\:{Z}_{i}\in\:{\mathbb{R}}^{{T}_{c}\times\:d}$ from the MK-CNN block (with $\:{d=\:d}_{model}$), a pointwise $\:1\times\:1$ convolution is first applied to mix channel-wise information across groups. Next, the attention computation begins by applying Layer Normalization (LN) in a pre-norm configuration, as defined in^53^:

$$\:{\stackrel{\sim}{Z}}_{i}=LN\left({Z}_{i}\right)$$

The normalized input $\:{\stackrel{\sim}{Z}}_{i}$ is then projected into query $\:\left(Q\right)$, key $\:\left(K\right)$, and value $\:\left(V\right)$ representations as follows:

$$\:{Q}_{h}={{\stackrel{\sim}{Z}}_{i}W}_{h}^{Q}\:\:\:\:\:\:\:\:\:\in\:{\mathbb{R}}^{{T}_{c}\times\:{d}_{k}},\:\:\:\:{\:\:\:\:\:W}_{h}^{Q}\in\:{\mathbb{R}}^{{d\times\:d}_{k}}$$

$$\:{K}_{g}={{\stackrel{\sim}{Z}}_{i}W}_{g}^{K}\:\:\:\:\:\:\:\:\:\in\:{\mathbb{R}}^{{T}_{c}\times\:{d}_{k}},\:\:\:\:{\:\:\:\:\:W}_{g}^{K}\in\:{\mathbb{R}}^{{d\times\:d}_{k}}$$

$$\:{V}_{g}={{\stackrel{\sim}{Z}}_{i}W}_{g}^{V}\:\:\:\:\:\:\:\:\:\in\:{\mathbb{R}}^{{T}_{c}\times\:{d}_{v}},\:\:\:\:{\:\:\:\:\:W}_{g}^{V}\in\:{\mathbb{R}}^{{d\times\:d}_{v}}$$

Here$\:,\:h\in\:\{1,...,H\}$ denotes the query head index, $\:g\in\:\{1,...,G\}$ denotes the key-value group index. All heads assigned to the same group $\:g$ share the same key and value projections $\:{K}_{g}$ and $\:{V}_{g}$, while maintaining distinct query projections $\:{Q}_{h}$​. This design enables each head to produce different attention distributions even when using the same key-value pair. The group assignment is defined by a mapping function $\:g\left(h\right)$, which associates each head $\:h$ with its corresponding group index. Assuming $\:H$ is divisible by $\:G$, the mapping is given by:

$$g\left( h \right) = 1 + \left\lfloor {\frac{{\left( {h - 1} \right)G}}{H}} \right\rfloor ,~~\;\;h = 1, \ldots ,H$$

The attention output for head $\:h$, using the shared key and value from its corresponding group $\:g\left(h\right)$, is computed as:

$$\:{GQA}_{h}\left({Q}_{h},\:{K}_{g\left(h\right)},\:{V}_{g\left(h\right)}\right)=\text{s}\text{o}\text{f}\text{t}\text{m}\text{a}\text{x}\left(\frac{{Q}_{h}\:{{K}_{g\left(h\right)}^{T}}_{\:}}{\sqrt{{d}_{k}}}\right){V}_{g\left(h\right)}\in\:{\mathbb{R}}^{{T}_{c}\times\:{d}_{v}}$$

The outputs from all heads are concatenated and passed through a linear projection:

$$\:GQA(Q,\:\:K,\:\:V)=\:\text{C}\text{o}\text{n}\text{c}\text{a}\text{t}\left({GQA}_{1},\:\dots\:,{GQA}_{H}\right){W}^{O}\in\:{\mathbb{R}}^{{T}_{c}\times\:d},\:\:\:{W}^{O}\in\:{\mathbb{R}}^{H{d}_{v}\times\:d}$$

A residual connection with a $\:\text{d}\text{r}\text{o}\text{p}\text{o}\text{u}\text{t}$ is added to produce the final layer output:

$$\:{O}_{i}={Z}_{i}+\text{D}\text{r}\text{o}\text{p}\text{o}\text{u}\text{t}\left(\text{G}QA\left(Q,\:K,\:V\right)\right)\:\:\:\:\:\:\in\:{\mathbb{R}}^{{T}_{c}\times\:d}$$

Following the GQA block, the output is passed through a position-wise FFN, which consists of two linear transformations with a Gaussian Error Linear Unit (GELU) activation in between. The sub-layer is applied in a pre-norm configuration, starting with layer normalization:

$$\:{\stackrel{\sim}{O}}_{i}=LN\left({O}_{i}\right)$$

The FFN is then defined as:

$$\:\text{F}\text{F}\text{N}\left({\stackrel{\sim}{O}}_{i}\right)=\text{D}\text{r}\text{o}\text{p}\text{o}\text{u}\text{t}\left({W}_{2}.\:\text{G}\text{E}\text{L}\text{U}\left({W}_{1}{\stackrel{\sim}{O}}_{i}\right)\right)\in\:{\mathbb{R}}^{{T}_{c}\times\:d},$$

where $\:{W}_{1}\in\:{\mathbb{R}}^{d\times\:rd}$, $\:{W}_{2}\in\:{\mathbb{R}}^{rd\times\:d}$, and $\:r\:$denotes the expansion ratio.

The GELU activation function is defined as:

$$\:GELU\left(x\right)=\:x.{\Phi\:}\left(x\right),\:\:where\:\varPhi\:\left(x\right)=\frac{1}{2}\left[1+erf\left(\frac{x}{\sqrt{2}}\right)\right]$$

Here, $\:{\Phi\:}\left(x\right)$ denotes the cumulative distribution function (CDF) of the standard Gaussian distribution, and$\:\:erf\left(x\right)$ is the Gauss error function.

Finally, a residual connection is added to complete the sub-layer:

$$\:{E}_{i}={O}_{i}+\text{F}\text{F}\text{N}\left({\stackrel{\sim}{O}}_{i}\right)\:\:\:\:\:\:\in\:{\mathbb{R}}^{{T}_{c}\times\:d}$$

To regularize training, a twofold strategy is employed. First, a standard dropout with a fixed rate of $\:{p}_{e}=0.$4 is applied after both the attention and FFN sublayers (as shown in the equations above). Second, stochastic depth is introduced through a layer-wise DropPath schedule, where the drop-path probability increases quadratically with layer depth (with $\:{\text{d}\text{r}\text{o}\text{p}\_\text{p}\text{a}\text{t}\text{h}\text{}}_{\text{m}\text{a}\text{x}}=0.25$). Specifically, the drop-path rate for the $\:{i}^{th}$ Transformer layer is defined as:

$$\:{drop\_path}_{i}={\left(\frac{i}{N-1}\right)}^{2}\cdot\:{drop\_path}_{max}$$

This combination of fixed dropout and depth-aware DropPath maintains stability in the early layers while enforcing stronger regularization in deeper ones, enabling the encoder to capture long-range temporal dependencies effectively without overfitting.

The Transformer encoder outputs a sequence of the same length $\:{T}_{c}$​, where each token embedding is enriched with contextual information from the entire trial. To generate the final feature representation, a pointwise $\:1\times\:1$ convolution is applied to reduce the dimensionality of the Transformer’s output from $\:d$ to $\:{d}_{group}$. The resulting sequence is then concatenated with the MK-CNN features $\:{Z}_{i}$, producing the model feature representation $\:{F}_{i}\in\:{\mathbb{R}}^{{T}_{c}\times\:{d}_{F}}$, where $\:{d}_{F}=\left({n}_{groups}+1\right){d}_{group}$. These features serve as enriched EEG representations suitable for downstream tasks. In the proposed architecture, a Temporal Convolutional Network is employed as the classification head, which takes $\:{F}_{i}$ as input and produces the final output logits.

### Temporal convolutional network

The final stage of the architecture is the TCN head, which processes the fused feature representation $\:{F}_{i}$, obtained from both the MK-CNN and Transformer encoder, to generate the classification output. TCNs utilize dilated convolutions to capture long-range temporal dependencies in time series data, while preserving causality, which is crucial for analyzing the temporal dynamics of EEG signals. The TCN head adopts a structure similar to the temporal convolutional block described in^31 ^with the following modification: the 1D convolution layers are configured with the argument $\:groups\:=\:{n}_{groups}+1$. This grouped convolution ensures that features from each group—originating either from the MK-CNN or the Transformer—are processed independently, without inter-group mixing.

**Fig. 6:**
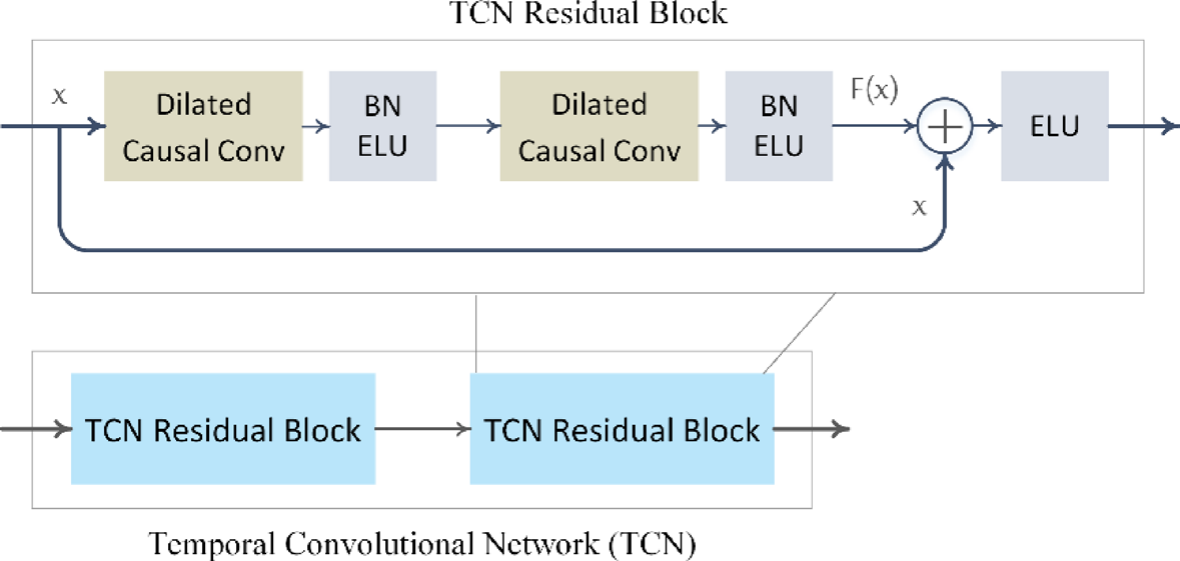
The temporal convolutional network.

The TCN used in the proposed architecture consists of $\:L=2$ residual blocks, each containing two dilated causal 1D convolutional layers with a kernel size of $\:{K}_{T}=4$ and the number of filters equal to the input dimensionality $\:{d}_{F}$. Each convolution is followed by batch normalization (BN)^54^ and an exponential linear unit (ELU) activation. A residual connection spans the entire block, as illustrated in (Fig. 6).

The receptive field size (RFS) of the TCN increases exponentially with the number of residual blocks $\:L$, due to the growing dilation rate in successive layers. It is determined by the number of blocks $\:L$ and the kernel size $\:{K}_{T}$, and is given by:

$$\:\text{R}\text{F}\text{S}=1+2\left({K}_{T}-1\right)\left({2}^{L}-1\right)$$

**Fig. 7:**
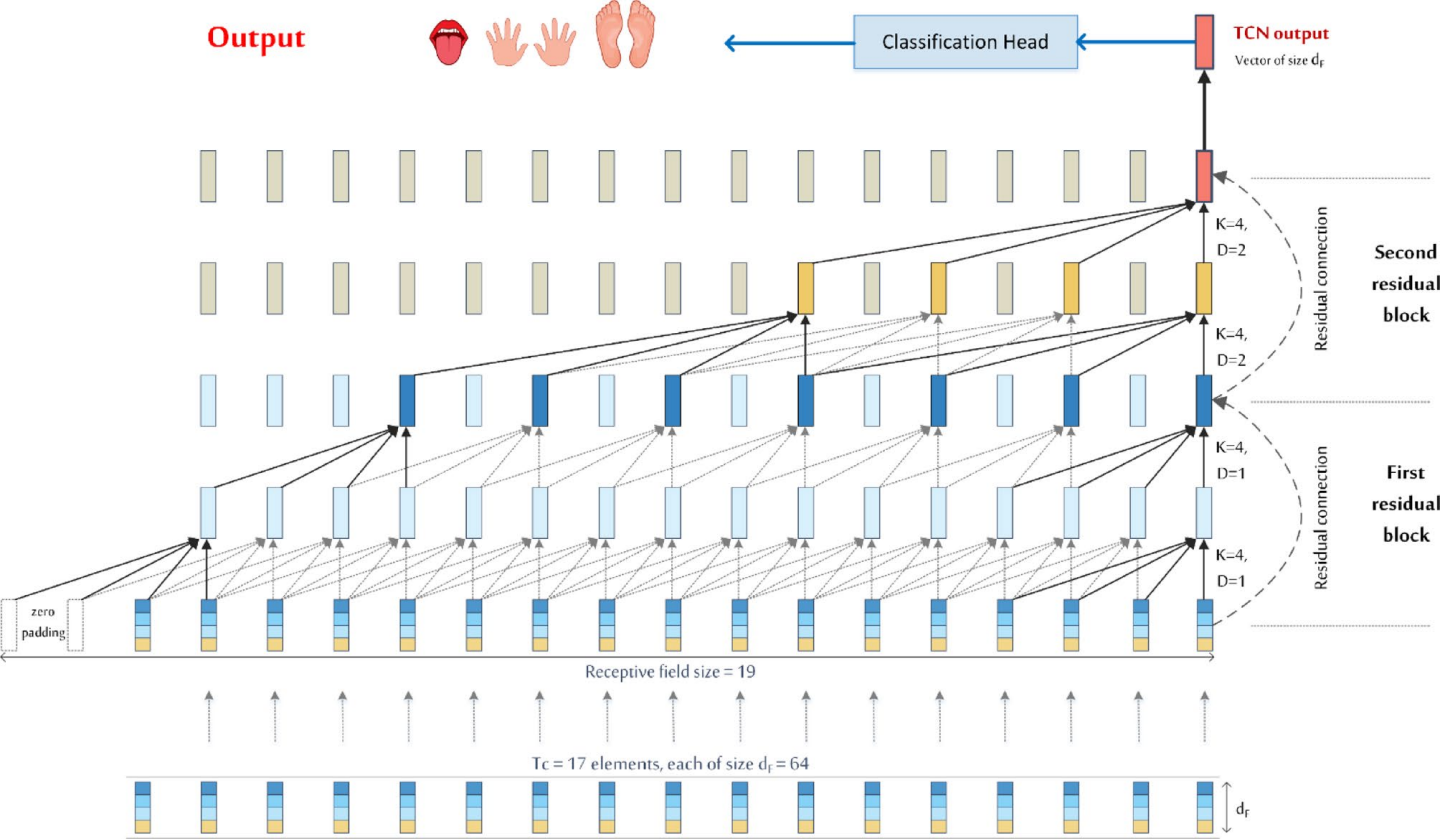
Temporal convolutional network (TCN) head: the feature sequence$\:\:{F}_{i}\in\:{\mathbb{R}}^{{T}_{c}\times\:{d}_{F}}$, generated by the MK-CNN and Transformer encoder (as shown in Fig. 2), is processed through two residual TCN blocks. Each block employs dilated causal convolutions with a kernel size of $\:{\text{K}}_{T}=4$ and dilation rates of $\:\text{D}=$1 and $\:\text{D}=2$, respectively, resulting in a total receptive field of 19 time steps. The output at the final time step—a vector of size $\:{d}_{F}$—is passed to a convolutional classification layer to produce the motor imagery (MI) class logits.

For example, with $\:L=2$ and $\:{K}_{T}=4$, the TCN achieves an RFS of 19, enabling it to capture dependencies across up to 19 time steps, as illustrated in (Fig. 7). To ensure complete temporal coverage without information loss, the input sequence length should be less than or equal to the $\:\text{R}\text{F}\text{S}$.

Figure 7 shows a TCN input sequence of length $\:{T}_{c}=17$, where each feature vector has a dimensionality of $\:{d}_{F}$. The TCN applies stacked causal convolutions to produce an output sequence of the same length. However, only the final output, corresponding to the last time step, is retained, as it summarizes information from the entire input. This final feature vector, of size $\:1\times\:{d}_{F}$, is passed to the classification head to produce the final prediction.

To map this feature vector to class predictions, a pointwise $\:1\times\:1$ convolution is applied within each group, reducing the dimensionality from $\:{d}_{group}\:$ to $\:{n}_{classes}$, where $\:{n}_{classes}$ corresponds to the number of motor imagery classes. This produces a matrix of logits with shape $\:{n}_{groups}\:\times\:{n}_{classes}$. The per-group logits are then averaged along the group dimension, yielding a single $\:{n}_{classes}$-dimensional output vector.

The entire TCFormer model is trained end-to-end by minimizing the cross-entropy loss between the predicted class logits and the ground-truth labels. The loss is defined as:

$$\:L=-\frac{1}{M}\sum\:_{i=1}^{M}\sum\:_{j=1}^{N}{y}_{ij}\text{l}\text{o}\text{g}\left({\widehat{y}}_{ij}\right)$$

Where $\:M$ is the number of training examples, $\:N$ is the number of classes, $\:{y}_{ij}$ is the ground truth label, and $\:{\widehat{y}}_{ij}$ is the predicted probability for class $\:j$ of sample *i*.

Unless otherwise specified, the hyperparameters used in all experiments are listed in (Table 1). These values were empirically determined through preliminary experimentation and remained fixed across all subjects and datasets.

**Table 1:** Hyperparameter configuration for TCFormer components: Transformer encoder, MK-CNN front-end, and TCN head. These values are applied consistently across all subjects and datasets.

MK-CNN	# of Temporal filters ($\:{F}_{1}$)	32	Transformer encoder	# of layers ($\:N$)	2
	Kernel size ($\:{K}_{C}$)	20, 32, 64		# of query heads ($\:H$)	4
	Depth multiplier (*D*)	2		# of key-value groups ($\:G$)	2
	1st pooling size (*P*_1_)	8		Dropout rate ($\:{p}_{e}$)	0.4
	2nd pooling size (*P*_2_)	7	TCN	# of residual blocks ($\:L$)	2
	Dropout rate ($\:{p}_{c}$)	0.4		Kernel size ($\:{K}_{T}$)	4
	Group dimension *(d*_*g*roup_)	16		Dropout rate ($\:{p}_{t}$)	0.3

## Experimental results and discussion

### Datasets

TCFormer is evaluated on three publicly available datasets, two EEG motor imagery datasets, BCI Competition IV-2a^55^ (BCIC IV-2a) and IV-2b^56^ (BCIC IV-2b), and one motor execution dataset, the High Gamma Dataset^41^ (HGD).

BCIC IV-2a: A standard 4-class MI dataset (left hand, right hand, feet, tongue) from 9 subjects, with 22 EEG channels (plus 3 EOG) at 250 Hz. Each subject performed 576 trials (2 sessions of 288). Each trial lasts eight seconds, with motor imagery performed during the middle four seconds. We follow the official evaluation protocol, using session 1 for training and session 2 for testing.

BCIC IV-2b: A 2-class MI dataset (left vs. right hand) recorded from 9 subjects using 3 EEG channels (C3, Cz, C4) at a sampling rate of 250 Hz. Each trial lasts 9 s, with motor imagery performed between the 3rd and 7th second in the first two sessions (4 s), and between the 3rd and 7.5th second in the last three sessions (4.5 s). The dataset comprises five sessions per subject, with 120 trials in sessions 1–2 and 160 trials in sessions 3–5. The first three sessions (400 trials) are used for training, and the last two sessions (320 trials) are reserved for testing.

HGD: The HGD is the largest dataset used in this study, comprising EEG recordings from 14 subjects with 128-channel EEG at 512 Hz. Subjects performed four classes of motor execution movements (not motor imagery, even though this dataset is used extensively and is sometimes referred to as a motor imagery dataset) of one of four classes: left hand, right hand, feet, or rest. As in the BCIC IV-2a dataset, each trial lasts 4 s. Following standard practice, we use 44 channels (motor cortex subset), apply a 4 Hz high-pass filter, and downsample to 250 Hz to maintain consistency with the BCIC IV-2a and IV-2a datasets. Each subject completed two recording sessions: the first session, which includes approximately 880 trials, is used for training, while the second session containing around 160 trials, is reserved for testing.

### Data augmentation

To mitigate overfitting that arises from the limited amount of labelled EEG available for training, we enlarge the training set using the segmentation and reconstruction (S&R) strategy as introduced by Lotte et al.^57^. This method operates in two stages:

1. Segmentation – Each motor imagery trial $\:{X}_{i}\in\:{\mathbb{R}}^{C\times\:T}$ is divided into $\:{N}_{s}$ non-overlapping temporal fragments of equal length (here $\:{N}_{s}=8$). This ensures that each fragment preserves the temporal structure within its original trial while capturing distinct portions of the $\:{\upmu\:}/{\upbeta\:}$ rhythm relevant for MI decoding.
2. Reconstruction – New synthetic trials are generated by randomly concatenating the $\:{N}_{s}$ fragments belonging only to trials of the same class, maintaining their original temporal order (e.g., fragments A–B–C–…–H). This recombination introduces intra-class variability while preserving label integrity and the natural causal flow of the EEG signal.

Let $\:\text{m}$ be the original number of trials and $\:{m}_{A}$​ the number of artificially generated trials; the augmented training set contains $\:m+{m}_{\text{A}}$ examples. In our experiments, we set $\:m={m}_{\text{A}}$, doubling the size of the training data. This approach introduces meaningful diversity in the temporal dynamics of motor imagery patterns and leads to consistent improvements in classification accuracy.

### Evaluation method and performance metrics

Two evaluation scenarios are considered: within-subject classification (subject-dependent) and cross-subject classification using a leave-one-subject-out (LOSO) approach (subject-independent). In both settings, a separate model is trained per target subject using the first session and evaluated on the second session (for BCIC IV-2b, this corresponds to the first three and last two sessions, respectively).

In the within-subject setting, the model is trained in the first session and evaluated in the second session of the same subject. This means both training and testing data come from the same individual, allowing us to assess the model’s performance in a subject-dependent scenario.

In contrast, the cross-subject LOSO setup assesses the model’s ability to generalize to unseen subjects^1^. Here, the model is trained using the first-session data from all subjects excluding the target subject, and then evaluated on the second-session data of the held-out subject. This process is repeated by rotating through all subjects.

The performance of both the proposed and baseline models is evaluated using two metrics: accuracy and Cohen’s kappa score. Specifically, the proposed models are assessed using the accuracy metric defined as:

$$\:\text{A}\text{C}\text{C}=\frac{\sum\:_{i=1}^{n}T{P}_{i}/{I}_{i}}{\:n}$$

Where *TP*_*i*_ denotes the number of true positives (correctly predicted samples) in class _*i*_, $\:{I}_{i}$​ is the total number of samples in class _*i*_, and $\:n$ is the total number of classes.

Cohen’s kappa score is calculated as:

$$\:\kappa\:\_score=\frac{1}{n}\sum\:_{a=1}^{n}\frac{{P}_{a}-{P}_{e}}{1-{P}_{e}}\:,\:\:\:\:$$

Where *P*_*a*_ is the observed agreement for class $\:a$, $\:{P}_{e}$​ is the expected agreement by chance, and $\:n$ is the number of classes.

Test accuracy and Cohen’s kappa score are reported using the final model checkpoint. Each model was trained independently for all subjects—9 in BCIC and 14 in HGD—using 5 random seeds per subject for BCIC and 3 for HGD, resulting in 45 and 42 training runs, respectively. For each subject, we compute the average accuracy and kappa across the multiple runs. The final reported results are then averaged across all subjects, with standard deviations reflecting the variability across runs. This repeated evaluation helps mitigate the effect of randomness during training, which is especially important given the limited size and high variability of EEG data.

To assess statistical significance, we perform a two-tailed paired t-test (α = 0.05) between TCFormer and each baseline. Comparisons are made using subject-level accuracy averages to avoid inflating the sample size due to repeated runs. To ensure fairness, statistical comparisons are conducted within the same augmentation setting: models trained without augmentation (–aug) are only compared to the –aug version of TCFormer, and those trained with augmentation (+ aug) are compared to the + aug version.

### Experimental setup

TCFormer was trained for a fixed number of epochs: 1000 for BCIC IV-2a and HGD, and 500 for BCIC IV-2b, using the Adam optimizer with a learning rate of 0.0009 in the within-subject setting. For the baseline models used in our comparison^24,29,31,34–36,40,41,58^ we follow the preprocessing steps and hyperparameter configurations reported in their respective papers. However, for all HGD experiments, a consistent 4 Hz high-pass filter is applied, regardless of the original setup. For EEGTCNet^29^ we use a 4-second input window (instead of the reported 4.5 s) and replace the ReLU activation with ELU, which yielded better performance. For CTNet^35^ we increase the number of temporal filters to F₁ = 20 (instead of 8), which improved accuracy compared to the original configuration. To enhance training stability and reduce loss oscillations, we apply a learning rate scheduler with a linear warm-up over the first 20 epochs, followed by a cosine decay schedule.

For cross-subject experiments, models are trained for 125 epochs on the BCIC datasets and 77 epochs on the HGD, reflecting the increased amount of training data. For example, in the BCIC IV datasets, the training set is approximately eight times larger than in the within-subject setting, as it includes data from multiple subjects. The warm-up phase is adjusted accordingly to 3 epochs.

All experiments were conducted on a workstation running Ubuntu 24.04.2 LTS, equipped with an Intel Xeon w5-3425 CPU, 125 GB RAM, and two NVIDIA RTX A6000 GPUs, each with 48 GB of VRAM. The software environment included Python 3.10, PyTorch 2.6.0, and CUDA 12.4. Complete training scripts and configuration files are included in the public code release to ensure full reproducibility.

### Ablation study

An ablation study was conducted to evaluate the individual contributions of each component within the TCFormer architecture. Three model variants were compared: (A) CNN-only: employs only the multi-kernel CNN module followed by a dense classification layer, excluding both the Transformer encoder and the TCN head. (B) CNN + TCN: includes the CNN module followed by a TCN head composed of two residual blocks and a classifier, representing a purely convolutional approach without self-attention. (C) Full TCFormer (CNN + Transformer + TCN): the complete architecture integrating all modules. All variants were trained under identical conditions on the BCIC IV-2a and IV-2b datasets to ensure a fair comparison. The results are summarized in (Table 2).

On BCIC IV-2a, the CNN-only baseline achieves 80.14% accuracy. Adding the TCN head (Variant B) raises performance to 83.26%, confirming the benefit of temporal convolutions in modeling the time-course structure of MI signals. Incorporating the Transformer (Variant C) further boosts accuracy to 84.79%, suggesting that global attention captures additional discriminative patterns. On BCIC IV-2b, the CNN-only and CNN + TCN variants perform similarly (~ 86.15%), indicating limited benefit from TCN alone. However, the full TCFormer achieves 87.71%, demonstrating the Transformer’s effectiveness in capturing global temporal dependencies and improving consistency. These results support the architectural design, showing that each component contributes to performance gains, and their combination achieves the highest accuracy.

### Analysis of the multi-kernel Convolution

To isolate the contribution of the MK-Conv front-end, a controlled grid search was conducted on the BCIC IV-2a dataset under the subject-dependent setting, following the same evaluation protocol used in previous ablation experiments. Each configuration reused the best-performing Transformer + TCN back-end while varying a single MK-Conv hyperparameter. All other settings were held constant, including three temporal kernels, 32 filters per kernel, a depthwise expansion factor of $\:\text{D}=2$, and the use of grouped attention. Table 3 reports the average test accuracy across nine subjects and the corresponding parameter count of the MK-Conv block. Note that the Transformer-TCN stack adds approximately 50.3k parameters, but this portion remains fixed across all variants.

Expanding the number of temporal kernels from one to three resulted in a notable accuracy improvement (+ 1.8%), confirming the value of multi-scale temporal receptive fields for decoding motor imagery rhythms. Increasing the number of filters per kernel ($\:{F}_{1}$) from 16 to 32 resulted in comparable accuracy but reduced performance variance, offering improved training stability. Beyond 32 filters, performance plateaued or declined, indicating over-parameterization. For the depthwise expansion factor ($\:D$), a moderate value ($\:D=2$) provided the best trade-off between capacity and generalization, while larger values ($\:D>4$) increased overfitting. Enabling grouped channel attention via a lightweight SE attention module added only ~ 0.2k parameters, yet improved accuracy by 0.35% and reduced variance. Overall, the baseline MK-Conv configuration—three kernels, 32 filters, $\:D=2$, and grouped attention enabled—offered the best balance between accuracy and efficiency, and was adopted in the final TCFormer architecture.

**Table 2:** Ablation results on BCIC IV-2a and BCIC IV-2b datasets, showing the impact of adding the Transformer encoder and TCN head. Variant A uses only the convolutional front-end and a dense classifier, yielding the lowest accuracy. Adding the TCN alone (Variant B) improves performance, highlighting the importance of Temporal context integration. The full TCFormer (Variant C) further improves accuracy over B, demonstrating that the Transformer’s global attention provides complementary benefits on top of the CNN + TCN.

Model variant	# Params	BCIC IV-2a		BCIC IV-2b	
		Accuracy (%)	Kappa	Accuracy (%)	Kappa
A. CNN-only (no Transformer, no TCN)	27.5 k	80.14 ± 0.69	0.74 ± 0.01	86.15 ± 1.14	0.72 ± 0.02
B. CNN + TCN (no Transformer)	37.3 k	83.26 ± 0.94	0.78 ± 0.01	86.16 ± 0.45	0.72 ± 0.01
C. CNN + Transformer + TCN (TCFormer)	77.8 k	84.79 ± 0.43	0.80 ± 0.01	87.71 ± 0.24	0.75 ± 0.01

**Table 3:** Ablation of MK-Conv hyperparameters on BCIC IV-2a (within-subject setting). Each variant modifies one MK-Conv component, with the Transformer + TCN back-end fixed. Results report mean accuracy ± standard deviation (across multiple runs) over nine subjects, along with MK-Conv parameter count.

Variant	# Params (MK-Conv)	Accuracy (%)	Observation
# of temporal kernels			
1 kernel (20)	8.5 k	83.01 ± 0.23	Cannot capture multi-scale rhythms.
2 kernels (20, 32)	17.6 k	84.25 ± 0.73	Gains from multi-scale features.
3 kernels *(baseline)* (20, 32, 64)	27.5 k	84.79 ± 0.43	Best trade-off.
Filters per kernel ($\:{F}_{1}$)			
16	21.7 k	84.81 ± 0.67	Slight gain; higher variance.
32 *(baseline)*	27.5 k	84.79 ± 0.43	Optimal balance.
64	39.1 k	84.46 ± 0.05	Marginal value.
128	62.3 k	83.47 ± 0.59	Overfitting.
Depthwise expansion ($\:D$)			
1	23.7 k	84.72 ± 0.58	Limited channel diversity.
2 *(baseline)*	27.5 k	84.79 ± 0.43	Optimal.
4	35.2 k	84.61 ± 0.31	Marginal value.
6	42.9 k	84.09 ± 0.31	Overfitting.
Group attention			
Disabled	27.3 k	84.44 ± 0.75	−0.4% for −0.2 k params.
Enabled *(baseline)*	27.5 k	84.79 ± 0.43	Helps focus salient channels.

### Overall classification performance

The classification performance of TCFormer and baseline models was evaluated using accuracy, Cohen’s kappa score, and model complexity (measured by parameter count), under both subject-dependent and subject-independent settings. Results are summarized in Tables 4, 5 and 6 for the BCIC IV-2a, BCIC IV-2b, and HGD datasets, respectively. TCFormer consistently achieves the highest mean accuracy across all datasets.

For the BCIC IV-2a dataset, TCFormer demonstrated superior performance compared to all baseline methods (Table 4), achieving an average accuracy of 84.8 ± 0.43% in the subject-dependent setting and 63% in the subject-independent setting. These results represent a clear improvement over widely used models such as EEGNet (+ 12.2%) and ShallowNet (+ 19.1%), as well as other CNN-based models like BaseNet (+ 6.2%) and EEGTCNet (+ 6%). TCFormer also outperforms recent CNN-Transformer models, including CTNet (+ 2.9%) and EEGConformer (+ 9.4%). Compared to ATCNet—a strong prior model achieving 83.4% ± 0.49 (subject-dependent) and 60.1% ± 1.87 (subject-independent)—TCFormer achieves improvements of 1.39% and 3%, respectively. Despite its high performance, TCFormer remains relatively compact with just 78k parameters, offering a favorable trade-off between performance and model complexity.

Across all models, data augmentation consistently improved accuracy in the within-subject setting (typically by 2–4%), while its impact in the cross-subject setting remained modest or occasionally negative. This indicates that the applied S&R augmentation strategy is more effective at refining subject-specific patterns than at improving generalization across subjects.

**Table 4:** Comparison of classification accuracy and cohen’s kappa coefficient, along with model complexity, for various methods on the BCIC IV-2a dataset under subject-dependent (within-subject) and subject-independent (cross-subject) settings. The best results are highlighted in bold, and the second-best results are underline﻿d. Statistical significance (shown for the accuracy metric) is assessed using a paired t-test across 9 subjects, comparing each model with TCFormer. A single asterisk (*) indicates a significant difference at *p* < 0.05, and a double asterisk (**) indicates a highly significant difference at *p* < 0.01. The “±” values denote the standard deviation across 5 random runs.

Model	#Params (k)	w/ or w/o Aug	Accuracy (%)		Kappa	
			within-subject	cross-subject	within-subject	cross-subject
EEGNet^24^	1.7		70.39 ± 0.48**	52.01 ± 1.22**	0.61 ± 0.007	0.36 ± 0.016
		+aug	72.62 ± 0.32**	52.03 ± 0.88**	0.64 ± 0.004	0.36 ± 0.012
ShallowNet^41^	44.6		60.5 ± 2.18**	48.83 ± 0.99**	0.47 ± 0.029	0.32 ± 0.013
		+aug	65.72 ± 1.32**	47.31 ± 1.06**	0.54 ± 0.018	0.3 ± 0.014
BaseNet^40^	3.7		76.45 ± 0.69**	57.82 ± 1.01	0.69 ± 0.009	0.44 ± 0.013
		+aug	78.58 ± 0.41*	56.89 ± 0.88*	0.71 ± 0.005	0.43 ± 0.012
EEGTCNet^29^	4.1		75.62 ± 1.04**	55.09 ± 1.13**	0.68 ± 0.014	0.4 ± 0.015
		+aug	78.82 ± 0.61**	55.99 ± 0.84**	0.72 ± 0.008	0.41 ± 0.011
TS-SEFFNet^58^	334.8		76.65 ± 0.58**	56.74 ± 0.83*	0.69 ± 0.008	0.42 ± 0.011
CTNet^35^	152.7		78.08 ± 1.28**	59.67 ± 2.04	0.71 ± 0.017	0.46 ± 0.027
		+aug	81.91 ± 0.36*	60.09 ± 0.94	0.76 ± 0.004	0.47 ± 0.013
MSCFormer^34^	150.7		75.25 ± 0.44**	52.04 ± 2.82**	0.67 ± 0.006	0.36 ± 0.038
		+aug	79.16 ± 2.08**	54.27 ± 1.52*	0.72 ± 0.028	0.39 ± 0.02
EEGConformer^36^	789.6		70.7 ± 1.72**	45.44 ± 0.95**	0.61 ± 0.023	0.27 ± 0.013
		+aug	75.39 ± 0.53**	45.59 ± 0.66**	0.67 ± 0.007	0.27 ± 0.009
ATCNet^31^	113.7		83.4 ± 0.49	60.05 ± 1.87	0.78 ± 0.007	0.47 ± 0.025
		+aug	83.78 ± 0.84	59.66 ± 1.27*	0.78 ± 0.011	0.46 ± 0.017
**TCFormer (Proposed)**	77.8		83.06 ± 0.54	62.44 ± 1.43	0.77 ± 0.007	0.5 ± 0.019
		+aug	**84.79 ± 0.43**	**63.00 ± 0.60**	**0.8 ± 0.006**	**0.51 ± 0.008**

**Table 5:** Comparison of classification accuracy and cohen’s kappa coefficient for various methods on the BCIC IV-2b dataset under subject-dependent (within-subject) and subject-independent (cross-subject) settings. The best results are highlighted in bold, and the second-best results are underlined. Statistical significance (shown for the accuracy metric) is assessed using a paired t-test across 9 subjects, comparing each model with TCFormer. A single asterisk (*) indicates a significant difference at *p* < 0.05, and a double asterisk (**) indicates a highly significant difference at *p* < 0.01. The “±” values denote the standard deviation across 5 random runs.

Model	w/ or w/o Aug	Accuracy (%)		Kappa	
		within-subject	cross-subject	within-subject	cross-subject
EEGNet^24^		82.8 ± 0.29**	77.67 ± 1.1	0.66 ± 0.006	0.55 ± 0.022
	+aug	83.65 ± 0.46**	77.89 ± 0.73*	0.67 ± 0.009	0.56 ± 0.015
ShallowNet^41^		79.12 ± 0.68**	74.5 ± 0.92	0.58 ± 0.014	0.49 ± 0.018
	+aug	81.45 ± 0.5**	75.58 ± 1.01*	0.63 ± 0.01	0.51 ± 0.02
BaseNet^40^		84.51 ± 0.48*	78.55 ± 0.61	0.69 ± 0.01	0.57 ± 0.012
	+aug	86.11 ± 0.4	78.61 ± 0.57	0.72 ± 0.008	0.57 ± 0.011
EEGTCNet^29^		85.54 ± 0.77*	78.82 ± 0.51*	0.71 ± 0.015	0.58 ± 0.01
	+aug	86.74 ± 0.12	80.56 ± 0.2	0.73 ± 0.002	0.61 ± 0.004
TS-SEFFNet^58^		84.18 ± 0.42**	77.82 ± 0.78**	0.68 ± 0.008	0.56 ± 0.016
CTNet^35^		86.81 ± 0.29	79.44 ± 0.6	0.74 ± 0.006	0.59 ± 0.012
	+aug	86.91 ± 0.29	80.29 ± 0.42	0.74 ± 0.006	0.61 ± 0.009
MSCFormer^34^		85.57 ± 0.22*	78.88 ± 0.78	0.71 ± 0.004	0.58 ± 0.016
	+aug	87.6 ± 0.39	79.2 ± 0.95*	0.75 ± 0.008	0.58 ± 0.019
EEGConformer^36^		79.46 ± 0.75*	73.44 ± 0.9*	0.59 ± 0.015	0.47 ± 0.018
	+aug	81.89 ± 0.45*	75.25 ± 0.34	0.64 ± 0.009	0.51 ± 0.007
ATCNet^31^		86.25 ± 0.59	80.29 ± 0.42	0.73 ± 0.012	0.61 ± 0.008
	+aug	86.26 ± 0.06*	80.94 ± 0.03	0.73 ± 0.001	0.62 ± 0.001
**TCFormer (proposed)**		87.11 ± 0.44	79.73 ± 0.53	0.74 ± 0.009	0.59 ± 0.011
	+aug	**87.71 ± 0.24**	**81.34 ± 0.25**	**0.75 ± 0.005**	**0.63 ± 0.005**

On the BCIC IV-2b dataset, TCFormer achieves the highest performance across both evaluation settings (Table 5), demonstrating superior decoding capability with a moderate parameter count. In the within-subject scenario, TCFormer achieves 87.71 ± 0.24% accuracy, slightly outperforming MSCFormer (+ 0.11%) and CTNet (+ 0.8%) with lower variance. In the more challenging subject-independent setting, TCFormer leads with 81.34 ± 0.25% accuracy, surpassing ATCNet (+ 0.4%) and EEGTCNet (+ 0.78%). Notably, EEGConformer—despite its large parameter count—underperforms relative to CNN-based baselines, highlighting that parameter scale alone does not guarantee performance. These results confirm that the TCFormer design provides the best trade-off between model complexity and classification performance.

Consistent with observations on the BCIC IV-2a dataset, data augmentation on BCIC IV-2b leads to noticeable gains in the within-subject setting (typically + 1–2%). Notably, its impact in the cross-subject setting is more pronounced than in IV-2a, suggesting that the S&R strategy, while primarily enhancing subject-specific decoding, can offer modest improvements in generalization under more consistent data distributions.

For the HGD dataset (Table 6), TCFormer achieved the highest performance in both evaluation settings, confirming its effectiveness on large-scale EEG data. In the within-subject setting, TCFormer attained 96.27 ± 0.84% accuracy, outperforming all other models. While EEGConformer was the second-best performer (94.67 ± 0.25%), TCFormer achieved a higher score despite using nearly 10× fewer parameters (78k vs. 790k). In the subject-independent setting—a more challenging scenario—most models exhibited substantial accuracy drops. ShallowNet, the best CNN-based baseline, reached 72.47 ± 0.94%, while EEGConformer and TS-SEFFNet followed closely at ~ 69.9%. Notably, when increasing TCFormer’s Transformer depth from *N* = 2 to *N* = 5 (with 131k parameters), its cross-subject accuracy improved to 72.83 ± 0.25%, surpassing all baselines, including ShallowNet. This highlights TCFormer’s strong generalization across subjects and its capacity to scale with minimal complexity overhead. The results further confirm that TCFormer achieves the best trade-off between model complexity and classification performance on this dataset.

Consistent with findings on BCIC IV-2a and IV-2b, data augmentation using the S&R strategy led to noticeable improvements in the within-subject setting but offered limited gains in the cross-subject scenario, further highlighting its strength in enhancing subject-specific pattern learning rather than cross-subject generalization.

### Results per subject

**Fig. 8:**
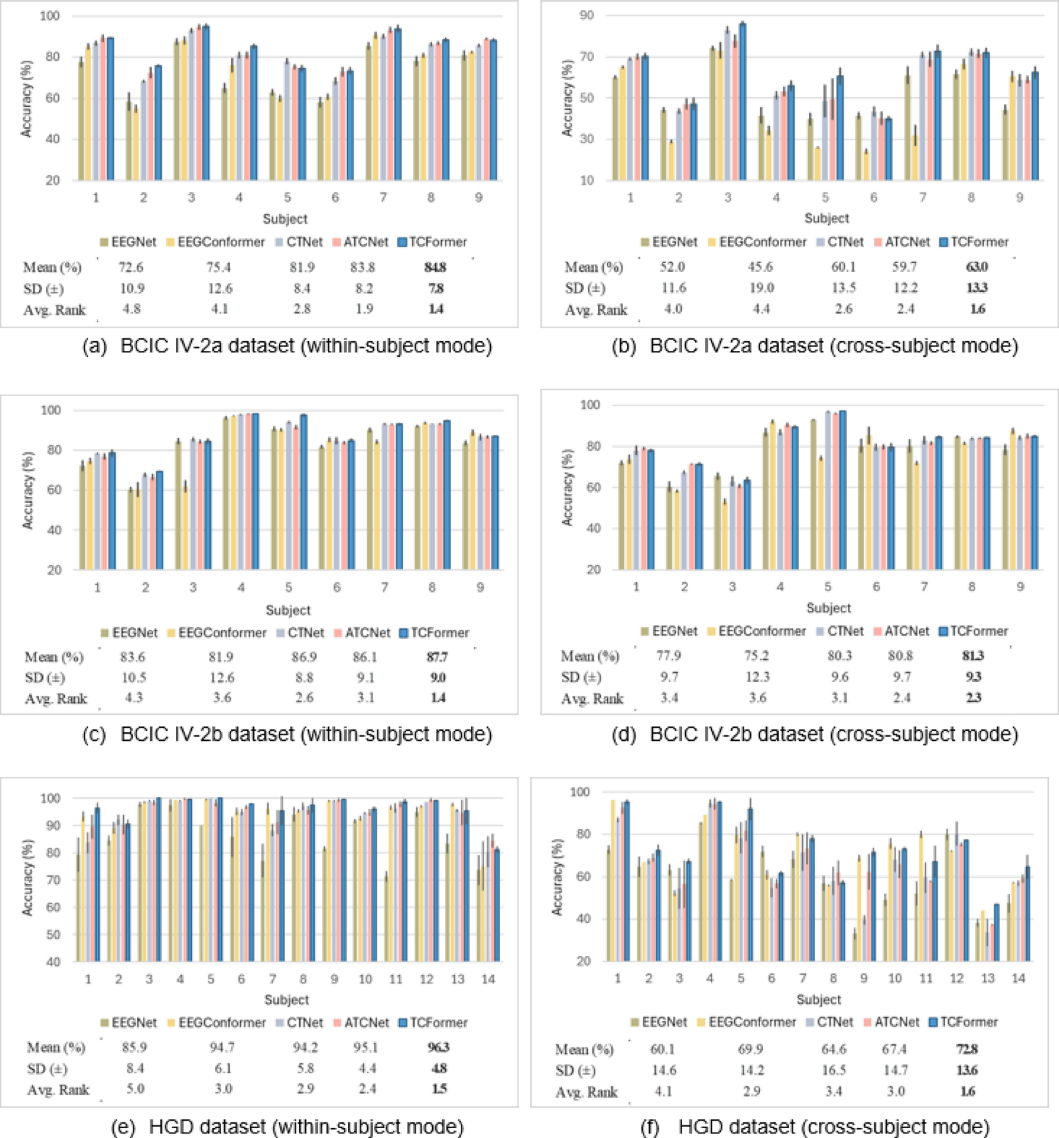
Subject-wise test accuracy (%) for three datasets under both within-subject and cross-subject evaluation modes. Panels display results for: (**a**) BCIC IV-2a (within-subject), (**b**) BCIC IV-2a (cross-subject), (**c**) BCIC IV-2b (within-subject), (**d**) BCIC IV-2b (cross-subject), (**e**) HGD (within-subject), and (**f**) HGD (cross-subject). Five models are compared—EEGNet, EEGConformer, CTNet, ATCNet, and the proposed TCFormer. Each bar represents the mean accuracy across multiple runs: five runs for BCIC datasets and three runs for HGD. The black error bars represent the standard deviation across these runs for each subject, reflecting variability in model performance. The tables below each panel summarize the mean accuracy, standard deviation, and average rank across subjects. TCFormer consistently achieves the highest mean accuracy and the lowest average rank, demonstrating superior performance across all datasets.

Tables 4, 5 and 6 report only the average performance across all subjects. However, evaluating the practical applicability of the models requires analyzing performance on a per-subject basis. To address this, Fig. 8 presents subject-wise results for the Transformer-based architectures alongside EEGNet, a representative non-transformer baseline, under both within-subject and cross-subject evaluation settings.

On the BCIC IV-2a dataset, TCFormer demonstrates strong performance in both within-subject and cross-subject evaluations. In the within-subject setting, it achieved the highest accuracy on 6 out of 9 subjects and remained highly competitive on the others. ATCNet ranked as the second-best model, achieving either the best or second-best accuracy on 8 out of 9 subjects. A similar pattern is observed in the cross-subject evaluation, where TCFormer also ranked first on 6 out of 9 subjects, with notable gains over other methods on S3 (+ 3.1%) and S5 (+ 11.2%), highlighting its strong generalization ability across subjects.

**Table 6:** Comparison of classification accuracy and cohen’s kappa coefficient, along with model complexity, for various methods on the HGD dataset under subject-dependent (within-subject) and subject-independent (cross-subject) settings. The best results are highlighted in bold, and the second-best results are underlined. The “±” values denote the standard deviation across 3 random runs.

Model	w/ or w/o Aug	Accuracy (%)		Kappa	
		Within-subject	Cross-subject	Within-subject	Cross-subject
EEGNet^24^		85.59 ± 0.45	57.95 ± 0.51	0.81 ± 0.006	0.44 ± 0.007
ShallowNet^41^		89.75 ± 0.54	72.47 ± 0.94	0.86 ± 0.007	0.63 ± 0.013
BaseNet^40^		93.64 ± 0.86	68.55 ± 1.74	0.92 ± 0.012	0.58 ± 0.023
EEGTCNet^29^		91.83 ± 1.8	60.59 ± 1.98	0.89 ± 0.024	0.48 ± 0.026
TS-SEFFNet^58^		92.45 ± 0.85	69.99 ± 0.57	0.9 ± 0.012	0.60 ± 0.008
CTNet^35^		93.53 ± 0.46	64.87 ± 1.48	0.91 ± 0.006	0.53 ± 0.02
	+aug	94.21 ± 0.52	64.6 ± 0.9	0.92 ± 0.007	0.53 ± 0.012
MSCFormer^34^		91.33 ± 0.35	61.06 ± 1.88	0.88 ± 0.005	0.48 ± 0.025
	+aug	94.31 ± 0.13	61.19 ± 1.96	0.92 ± 0.002	0.48 ± 0.027
EEGConformer^36^		93.6 ± 0.84	69.21 ± 0.57	0.92 ± 0.011	0.59 ± 0
	+aug	94.67 ± 0.25	69.92 ± 0.38	0.93 ± 0.004	0.6 ± 0.005
ATCNet^31^		93.65 ± 0.33	67.42 ± 0.24	0.92 ± 0.005	0.57 ± 0.003
**TCFormer (proposed)**		95.62 ± 0.84	69.89 ± 1.72	0.94 ± 0.011	0.6 ± 0.023
	+aug	**96.27 ± 0.84**	69.69 ± 2.61	**0.95 ± 0.012**	0.6 ± 0.035
	+aug, $\:N=5$	95.79 ± 0.51	**72.83 ± 0.25**	0.94 ± 0.007	**0.64 ± 0.004**

**Fig. 9:**
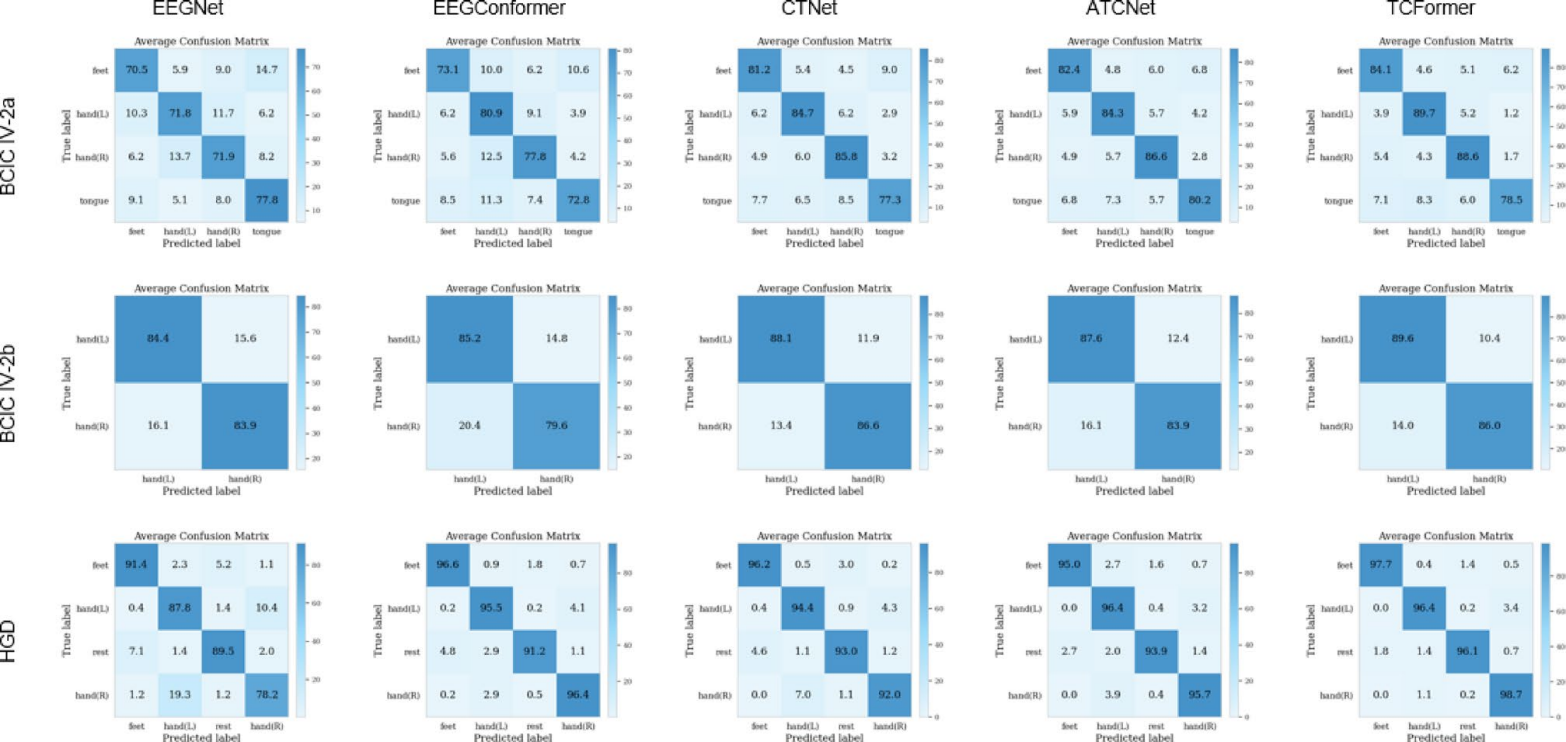
Average confusion matrices for five models—EEGNet, EEGConformer, CTNet, ATCNet, and the proposed TCFormer—on three EEG motor-imagery datasets: BCIC IV-2a (four‐class MI: feet, left hand, right hand, tongue), BCIC IV-2b (two‐class MI: left hand vs. right hand), and HGD (four‐class movement: feet, left hand, rest, right hand). The matrices correspond to the best-performing run for each model, averaged across all subjects. Darker diagonal entries indicate higher per‐class accuracy and overall model performance.

On the BCIC IV-2b dataset, TCFormer achieved the highest overall average accuracy; however, the performance margin over competing models was relatively narrow, especially in the cross-subject setting. In the within-subject evaluation, TCFormer attained the highest accuracy on 6 out of 9 subjects and remained competitive on the others, as illustrated in (Fig. 8). In the cross-subject setting, performance varied more noticeably across subjects—TCFormer ranked first on 3 out of 9 subjects while delivering comparable results on the rest. Some models outperformed TCFormer in specific cases—for instance, EEGNet led on S3 and S8, ATCNet on S1, and EEGConformer on S4, S6, and S9. These findings suggest that while TCFormer maintains consistently strong performance, its relative advantage diminishes on smaller datasets with fewer EEG channels.

For the HGD dataset, TCFormer demonstrated strong per-subject performance, particularly in the within-subject analysis where it frequently achieved the highest accuracy across most subjects, with scores often exceeding 95%. In the cross-subject setting, TCFormer continued to demonstrate strong generalization, achieving the highest accuracy in 7 out of 14 subjects, with notable margins on S3 (+ 4.1%), S5 (+ 10.2%), and S14 (+ 5.5%). Some inter-subject variability was still present, as EEGConformer outperformed TCFormer on S1, S7, S10, and S11. Although TCFormer achieved the highest score on S13, its accuracy dropped to 46.9%, highlighting the continued challenge of consistent cross-subject generalization in MI-EEG decoding.

Figure 9 presents the average confusion matrices across BCIC IV-2a, BCIC IV-2b, and HGD datasets, illustrating that TCFormer consistently achieves superior class-wise separation compared to baseline models. In BCIC IV-2a, TCFormer reduces misclassifications between overlapping motor imagery classes such as *tongue* and *hands*, achieving clearer boundaries and higher precision, particularly for *hand (L)* and *hand (R)*. In BCIC IV-2b’s binary setting, it minimizes cross-hand confusion and attains the cleanest class split. On the HGD dataset, where all models perform strongly, TCFormer further improves per-class accuracy—especially for *feet* and *rest*—while almost eliminating confusion between *hand* classes. These results highlight TCFormer’s ability to produce more discriminative and reliable predictions across varied EEG decoding tasks.

Overall, TCFormer exhibits the best balance of accuracy and efficiency, leveraging data augmentation, multi-scale convolution, and transformer encoder to achieve reliable performance across both subject-dependent and subject-independent settings. Its consistent results on the BCIC IV-2a, BCIC IV-2b, and HGD datasets confirm its effectiveness not only for motor imagery tasks but also for motor execution tasks, highlighting its broad applicability to diverse EEG decoding scenarios.

### Limitations

TCFormer demonstrates strong performance, but several limitations remain. Classification accuracy drops significantly in the cross-subject (LOSO) setting compared to within-subject evaluation across all datasets, reflecting the well-known distribution shift between training (source) subjects and unseen (target) subjects. This challenge, observed across all models, highlights the need for domain adaptation or subject-invariant representation learning to improve generalizability in real-world BCI applications. Additionally, performance variability among certain individuals in the LOSO setting indicates that adaptive normalization may help reduce subject-specific variance.

Another limitation lies in the computational overhead introduced by the dilated TCN head. While the TCN block significantly contributes to performance—as shown in the ablation study—it increases training time considerably compared to the MK-CNN and Transformer encoder. The TCN plays a key role in extracting temporal patterns not captured by the Transformer alone, suggesting two directions for future work: (1) optimizing the TCN module for faster training or (2) enhancing the Transformer encoder to effectively capture both short- and long-range temporal dependencies, potentially eliminating the need for a separate temporal head.

## Conclusion

This paper introduced TCFormer, a temporal convolutional Transformer for EEG-based motor imagery decoding. TCFormer integrates a multi-kernel convolutional front-end, an efficient Transformer encoder featuring grouped-query attention and rotary positional encoding, and a temporal convolutional network back-end. This design leverages the complementary strengths of CNNs (local feature extraction), Transformers (global context modeling), and TCNs (long-range temporal pattern learning) to achieve superior BCI performance. Extensive experiments on three benchmark datasets—BCIC IV-2a, BCIC IV-2b, and HGD—demonstrate that TCFormer consistently outperforms recent state-of-the-art models, achieving within-subject accuracies of 84.79, 87.71, and 96.27%, and cross-subject accuracies of 63.0, 81.34, and 72.83%, respectively. Detailed ablation studies confirm that each component meaningfully contributes to the model’s overall effectiveness. Notably, our integrated approach, which combines the Transformer encoder with CNN and TCN modules, significantly outperforms the more straightforward strategy of placing Transformer layers directly after CNN blocks, highlighting the importance of synergistic architectural design. Furthermore, the use of grouped-query attention demonstrates that efficiency improvements in Transformers can be successfully applied to neuroscientific data without compromising accuracy.

## Data Availability

All datasets used in this study are publicly available. The BCIC IV-2a and BCIC IV-2b motor imagery EEG datasets can be downloaded from the BCI Competition IV repository: http://www.bbci.de/competition/iv/. The High-Gamma Dataset (HGD) is available at: https://github.com/robintibor/high-gamma-dataset. Preprocessing scripts, trained TCFormer models, and other code supporting the findings of this study are provided in the following repository: https://github.com/altaheri/TCFormer.
